# Complete chloroplast genome of *Alternanthera philoxeroides* by *de novo* sequencing

**DOI:** 10.1080/23802359.2021.1931512

**Published:** 2021-06-04

**Authors:** Ping Jiang, Guangpei Xu, Yanfei He, Taotao Sun, Changli Liu, Cunwu Chen, Ruihua Zuo, Chuanbo Sun

**Affiliations:** a College of Biological and Pharmaceutical Engineering, West Anhui University, Lu’an, P. R. China; b Anhui Engineering Laboratory for Conservation and Sustainable Utilization of Traditional Chinese Medicine Resources, Lu’an, P. R. China

**Keywords:** *Alternanthera philoxeroides*, phylogenetic tree, chloroplast genome

## Abstract

*Alternanthera philoxeroides* (Mart.) *Griseb.* (*Alternanthera philoxeroides*) is an important herbage species, which could provide high-quality feed for livestock and poultry breeding. This paper is the first to report the *A. philoxeroides’s* chloroplast genomes, which were detected by *de novo* sequencing. The results showed that the length of *A. philoxeroides’* chloroplast genome sequence was 152,255 bp, including a large single-copy (LSC) region (84,670 bp), a small single-copy (SSC) region (17,343 bp), and two inverted repeat (IR) regions (25,121 bp). *Alternanthera philoxeroides’* chloroplast genome encoded 132 genes including 8 rRNA, 38 tRNA, and 86 protein-coding genes. After phylogenetic and cluster analysis, *A. philoxeroides* was closest to *Amaranthaceae*, and the relationship between *Amaranthus* and *Achyranthes* was closest.

*Alternanthera philoxeroides* (Mart.) *Griseb.* (*Alternanthera philoxeroides*) is an *Amaranthaceae* family originating in South America, and it is all over the world now (Telesnicki et al. 2011; Jin et al. 2021). *Alternanthera philoxeroides* has enlisted the Preliminary list of alien invasive species by the Ministry of Ecology and Environment of the People’s Republic of China in 2002. Although *Alternanthera philoxeroides* are a harmful species that invasive to China, it is used as herbage for animals’ breeding at present (Pan et al. 2007; Tan and Zhang 2007; Wang and Huang 2011). *Alternanthera philoxeroides* is very rich in wild resources spreading over the marshes, rivers, and lakes in China (Li et al. 2012; Yan et al. 2020). Thus it is a very promising industry to make rational use of the resources of *Alternanthera philoxeroides* to provide high-quality feed for livestock and poultry breeding. However, there is little research on the resource utilization of *Alternanthera philoxeroides,* especially the comprehensive utilization and development of its genetic resources and species resources. Since the chloroplast genome has conserved structure and orthologous comparing with the nuclear genome, it plays a pivotal role in *Alternanthera philoxeroides’* genetics and evolution (Cui et al. 2006; Zuo et al. 2019; Yinran et al. 2020). Nevertheless, there is no relevant chloroplast genome research about the *Alternanthera philoxeroides* leading the process of molecular genetics research was been prevented. Therefore, the complete chloroplast genomes of *Alternanthera philoxeroides* was been detected by *de novo* sequencing.

The fresh leaves of *Alternanthera philoxeroides* was been collected from Pihe National Wetland Park, Lu’an city, Anhui province, P. R. China (N:31.76°, E:116.49°), and the fresh leaves of *Alternanthera philoxeroides* was deposited at West Anhui University (Ping Jiang, 02000137@wxc.edu.cn) under the voucher number TCVM202008280035. The Plant DNA extraction kit was used to extract the total DNA of *Alternanthera philoxeroides.* After the DNA quality meets the sequencing requirements detected by the method of micro-volume spectrophotometer and 1% agarose electrophoresis, the DNA was escorted to Beijing Zhongxingbomai Technology for sequencing using Illumina NovaSep platform.

The Raw data were filtered and Get Organelle pipeline (Jin et al. 2020) was run to obtain high-quality data. The contig sequence was been assembled by SPAdes *de novo* software (Luo et al. 2012; Yinran et al. 2020), and the relative position of the genome in the contig sequences was acquired by the BLAT (Kent 2002) referencing the NCBI database (NC 024157.1, NC 011942.1, NC 009618.1, NC 000932.1, KX 352464.1). The Bandage tool (Wick et al. 2015) and the Geseq program (Tillich et al. 2017) were run to obtain the full-length frame diagram and annotate the chloroplast genome, respectively. The OGDRAW software (Lohse et al. 2013) was run to draw the physical map (GenBank accession number: MW285080).

Similar to other higher plants, the *Alternanthera philoxeroides* has a typical four-segment structure, including a large single-copy (LSC) region (84,670 bp), a small single-copy (SSC) region (17,343 bp), and two inverted repeat (IR) regions (25,121 bp). And the plastome sequence of *Alternanthera philoxeroides’* chloroplast genome was 152,255 bp. Its GC content was 36.40% and contained 132 genes including 8 rRNA, 38 tRNA, and 86 protein-coding genes. To determine the phylogenetic status of *Alternanthera philoxeroides* in *Amaranthaceae* plants, we used two *Amaranthus*, two *Achyranthes*, three *Chenopodium,* and three *Fagopyrum* plants and 1 outgroup (*Silene*) in NCBI for phylogenetic analysis. The GTRCATX model from raxmlGUI version 1.5 b (Silvestro and Michalak 2012) performed 1000 bootstrap replicates to calculate the evolutionary relationship and drawing the phylogenetic tree. The cluster analysis results showed that *Alternanthera philoxeroides* was closest to *Amaranthaceae*, and the relationship between *Amaranthus* and *Achyranthes* was closest (Figure 1).

**Figure 1.**
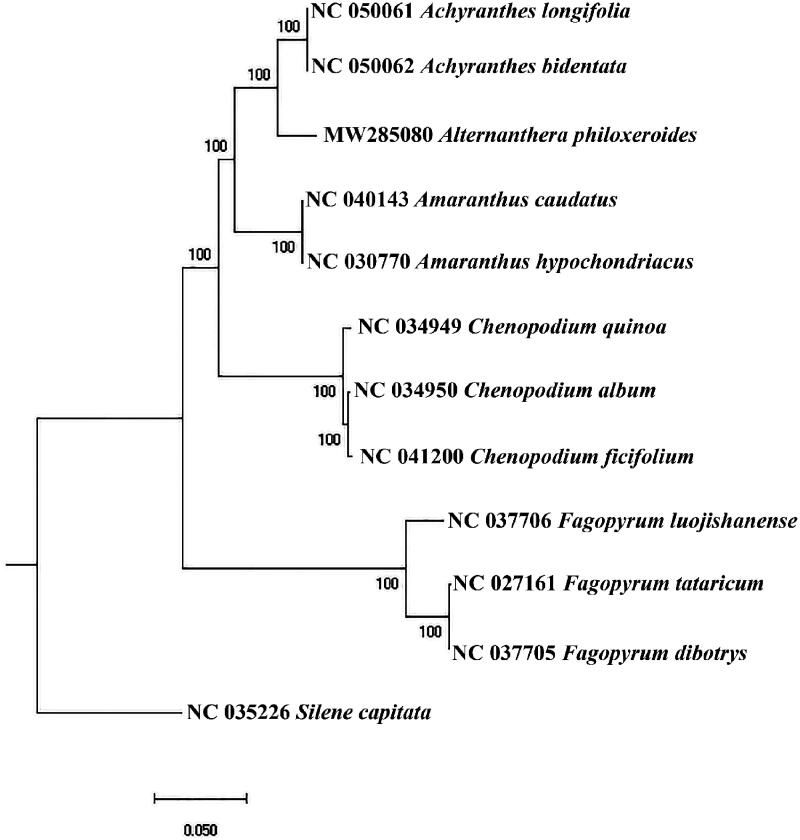
the phylogenetic tree.

## Data Availability

The data that support the findings of this study have send it up to BankIt (2403319) of National Center for Biotechnology Information, and provided GenBank accession number (MW285080). The associated BioProject and Bio-Sample numbers are PRJNA683712 (https://www.ncbi.nlm.nih.gov/bioproject/PRJNA683712) and SRX9689754 (https://www.ncbi.nlm.nih.gov/sra/SRX9689754]), respectively.
